# KLF7 induced ADRB3-dependent IL-6 production in brown adipocytes during stress

**DOI:** 10.1016/j.jlr.2026.100981

**Published:** 2026-01-19

**Authors:** Maodi Liang, Meixiu Zhang, Yanting Hou, Fangyuan Yuan, Huizi Zhang, Mengyuan Zhao, Lili Xu, Qin Liu, Yurui Su, Xiaolong Chu, Wei Li, Jingzhou Wang, Jianxin Xie, Cuizhe Wang, Qinghua Cui, Jun Zhang

**Affiliations:** 1 Department of Medical Genetics, Shihezi University School of Medicine, Shihezi, Xinjiang Province, China; 2 Department of Obstetrics, Shaanxi Provincial People’s Hospital, Xi'an, Shaanxi Province, China; 3 Department of Medical Genetics, Medical College of Tarim University, Xinjiang Province, China; 4 School of Sports Medicine, Wuhan Institute of Physical Education, Wuhan, Hubei Province, China

**Keywords:** KLF7, IL-6, ADRB3, stress, liver gluconeogenesis

## Abstract

In recent studies, acute physiological stress has been shown to enhance liver gluconeogenesis by activating β3-adrenergic receptor (ADRB3)-dependent interleukin-6 (IL-6) production in brown adipocytes, effectively fueling “fight or flight” responses. However, the specific molecular mechanism of this IL-6 production in an ADRB3-dependent manner is not fully understood. ADRB3 regulates multiple metabolic programs in adipose tissue, including thermogenesis, lipolysis, and glucose uptake, by activating cAMP-PKA-CREB signaling. Our previous studies revealed that the transcription factor Krüppel-like factor 7 (KLF7) transcriptionally induces *IL-6* expression in white adipocytes. Using *Klf7*-adipocyte knockout mice, we showed that *Klf7* is also required for ADRB3-induced IL-6 production during stress. cAMP-PKA-CREB signaling mediates this transduction via stress and ADRB3 agonist administration in a mouse model in vivo, as well as in brown adipocytes cultured in vitro. cAMP response element-binding protein (CREB) positively regulates *KLF7* transcription by binding to the promoter of *KLF7*. These findings indicate that stress-induced IL-6 production is dependent on *Klf7* in adipocytes. *KLF7*, as a target gene of CREB, responds to ADRB3 activation to increase endocrine IL-6 in a cAMP-PKA-CREB signaling-dependent manner. Our study provides a new theoretical basis for elucidating and enriching the novel mechanism of stress-induced IL-6 production in brown adipocytes.

## Graphical abstract

KLF7 responds to ADRB3 activation in brown adipocytes to increase endocrine IL-6 in a cAMP-PKA-CREB signaling-dependent manner.

**Figure:**
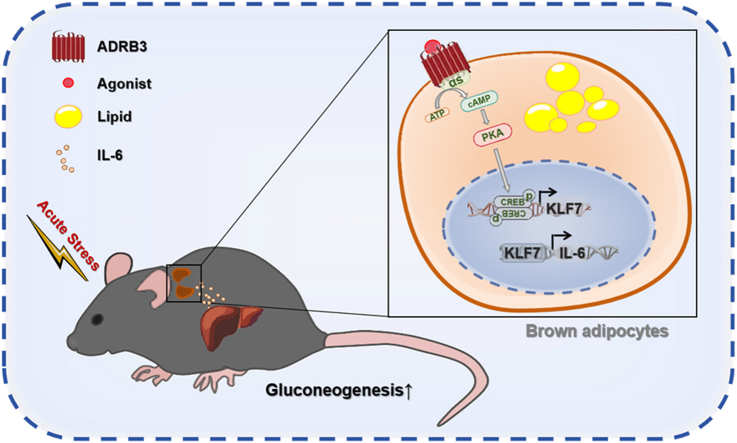

Brown adipose tissue (BAT) has been extensively investigated as a promising therapeutic target for obesity because of its specialized thermogenic programs that block mitochondrial adenosine triphosphate (ATP) synthesis through uncoupling protein 1 (UCP1)-dependent uncoupling of oxidative phosphorylation (1, 2). Many exogenous stimuli, such as cold exposure and ADRB3 agonists, induce nonshivering thermogenesis in BAT and promote weight loss (3, 4). Consequently, extensive research endeavors over the past decade have been devoted to alleviating obesity-related metabolic disorders through “burning” BAT.

In 2020, a study published in the journal Cell authored by Qing *et al.* revealed novel insight into BAT extending beyond thermogenesis. BAT serves as a stress-responsive endocrine organ that releases interleukin-6 (IL-6) into the circulation in an ADRB3-dependent manner. IL-6 enhances hepatic gluconeogenesis by binding to the IL-6 receptor (IL-6R) in the liver, a metabolic adaptation that fuels the "fight or flight" response (5). However, the mechanism underlying ADRB3-induced IL-6 production in brown adipocytes during stress has not been well studied.

Krüppel-like factor 7 (KLF7), a member of the zinc-finger transcription factor subfamily, plays significant regulatory roles in neurogenesis, adipogenesis, type 2 diabetes, hematologic diseases, and the progression of cancers (6, 7, 8, 9, 10). Our previous studies revealed that KLF7 specifically interacts with the promoter of *IL-6* and transcriptionally activates its expression, exacerbating the inflammatory response in the white adipose tissue of mouse models of obesity (11, 12, 13). Nevertheless, the involvement of KLF7 in ADRB3-mediated IL-6 release from brown adipocytes during stress remains poorly understood.

ADRB3 functions as a G protein–coupled receptor through binding to the Gα and Gβγ subunits. G protein–coupled receptors transduce intracellular signals by coupling to G protein family members, including Gas, Gai/o, Gaq, and G12/13 (14, 15, 16). Studies have shown that ADRB3 couples to Gαs to activate adenyl cyclase (AC) in adipocytes, leading to an increase in intracellular cyclic adenosine monophosphate (cAMP) levels, which directly activates protein kinase A (PKA) to phosphorylate cAMP response element-binding protein (CREB). Activated CREB binds to cAMP response element (CRE) sites within the promoter region of target genes, regulating thermogenesis, lipolysis, and glucose transport in adipose tissue (17, 18). How the crosstalk between cAMP-PKA-CREB signaling and KLF7 results in ADRB3-induced IL-6 production during stress remains largely unexplored.

Here, we found that stress-induced IL-6 production is dependent on *Klf7* in adipocytes. *KLF7*, as a target gene of CREB, responds to ADRB3 activation in adipocytes to increase endocrine IL-6 in a cAMP-PKA-CREB signaling-dependent manner. In addition, adipocyte-derived IL-6 contributes to the regulation of hepatic gluconeogenic gene expression. Our study therefore mechanistically reveals that stress-induced ADRB3 activation increases IL-6 release from adipocytes via the cAMP-PKA-CREB-KLF7 pathway, thereby promoting hepatic gluconeogenesis.

## Materials and Methods

### Mice

Six-to eight-week-old male C57BL/6J mice were obtained from Hunan SJA Laboratory Animal Co., Ltd. and housed at the animal facility of Shihezi University School of Medicine. KLF7flox/flox mice with Adipoq-Cre transgenic mice were purchased from Cyagen Biosciences Inc. Adipocyte-specific *Klf7* knockout (AKO) mice were generated by crossing *Klf7*^flox/flox^ mice with Adipoq-Cre transgenic mice. Littermate *Klf7*^flox/flox (Cre–)^ mice were used as wild-type (WT) controls. Genotyping was performed by PCR using tail DNA. Mice were housed under a 12-h light/12-h dark cycle with ad libitum access to standard chow and water. All experimental animals were approved by the First Affiliated Hospital of Shihezi University and subjected to regulatory review (No. A2019-086-01).

For the acute stress mouse model, as described in previous studies (5), retroorbital bleeding was performed using a hematocrit capillary four hours before the experiment, after which blood was collected from the contralateral eye for further analysis.

For the compound-treated mice, CL316,243 (1 mg/kg; Abcam, 151126-84-0) was injected intraperitoneally, and tissues and blood were collected 2 h after injection. Saline was used as the vehicle control for CL316,243. H89 (5 mg/kg; MCE, HY-15979) was also injected i.p. 1 h before stress or CL316,243 injection and was injected every 2 h during the experiment. The vehicle control for H89 consisted of saline containing 5% DMSO, matched in volume and injection schedule to the inhibitor-treated groups.

### Cell culture and transfection

Primary brown adipocytes were isolated from the interscapular BAT of 2–4 week-old C57BL/6J mice and cultured in DMEM/F12 (1:1; Gibco, 11330032) supplemented with 10% FBS (BioMed, C04001, China) and 1% P/S (Solarbio, P1400, China). C3H10T1/2 cells and Hepa 1–6 cells were obtained from ATCC and cultured in DMEM (BioMed, C3103, China) supplemented with 10% FBS and 1% P/S. For brown adipocyte differentiation, when the primary brown adipocytes and C3H10T1/2 cells reached full confluence, they were cultured for an additional 48 h. Next, the culture medium was supplemented with insulin (850 nM; MCE, HY-P0035), dexamethasone (2 μg/ml; MCE, HY-14648), indomethacin (125 nM; MCE, HY-14397), 3-isobutyl-1-methylxanthine (0.5 mM; MCE, HY-12318), triiodothyronine (1 nM; MCE, HY-A0070A) and rosiglitazone (0.5 μM; MCE, HY-17386) for 48 h, followed by a 6-day culture period with insulin, dexamethasone, and rosiglitazone.

For stimulation or inhibition experiments, differentiated brown adipocytes were treated as follows: the ADRB3 agonist CL316,243 was used at 5 μM for 4 h; the adenylate cyclase activator forskolin was applied at 10 μM for 4 h. Where indicated, cells were pretreated for 1 h with the PKA inhibitor H89 (10 μM) or the CREB inhibitor 666-15 (10 μM) prior to the addition of CL316,243 or forskolin.

The CREB plasmid and the pGL-3 basic-*KLF7* promoter plasmid were obtained from Beijing SyngenTech Co., Ltd. The plasmid volume was calculated on the basis of the concentrations of the CREB (2 μg/ml) and pGL-3 basic-*KLF7* promoter plasmids (3 μg/ml). The plasmid was transfected into HEK-293T cells in DMEM containing Lipo2000 (Thermo Fisher Scientific, 11668019) for 4 h, after which the culture medium was replaced with FBS-containing DMEM.

### Western blotting

Fresh tissue and cell samples were collected for protein extraction using RIPA buffer (Solarbio, R0010, China) containing PMSF (Solarbio, P0100, China) and a phosphatase inhibitor cocktail (Proteintech, PR20015, China). The homogenate was centrifuged at 4°C and 12,000 rpm for 15 min, and the supernatant was collected for BCA quantification and standardization. Then, the protein lysates were mixed with 4× SDS‒PAGE loading buffer with DTT (Solarbio, P1015, China) and denatured at 100°C. Protein samples were separated by SDS‒PAGE and transferred to PVDF membranes (Millipore, P005505). The membranes were soaked in protein-free fast blocking buffer (Servicebio, G2052, China) for 10 min and then incubated overnight at 4°C with primary antibodies against KLF7 (Abcam, ab197690), IL-6 (Abcam, ab290735), phospho-PKA substrate (CST, 9624), p-CREB (Proteintech, 28792-1-AP, China), CREB (Proteintech, 12208-1-AP, China), β-Tubulin (ZSGB-BIO, TA-10, China), and UCP1 (Abcam, ab234430), followed by a 2-h incubation with goat anti-rabbit immunoglobulin G (IgG) (ZSGB-BIO, ZB-2301, China) and goat anti-mouse IgG (ZSGB-BIO, ZB-2305, China) secondary antibodies at room temperature. Finally, imaging was performed after incubating the membranes in chemiluminescent solution (Millipore, P90720).

### Quantitative real-time PCR analysis

Tissues or cells were fully lysed in TRIzol reagent (Invitrogen) to extract total RNA. The extracted total RNA was reverse transcribed into complementary DNA (cDNA) using a kit (Thermo Fisher Scientific, K1622) following the protocol provided by the manufacturer. cDNA was quantified, and the PCR system was prepared according to the instructions provided with a kit. Real-time quantitative PCR analysis of the cDNA was performed using the SYBR Green probe. Relative gene expression values were calculated using the 2^−ΔΔCt^ method with β-actin as the endogenous control. The sequences of the primer pairs are provided in Table 1.

**Table 1:** Primer for qRT-PCR

Gene	Forward	Reverse
Mus-*Klf7*	TCCACGACACCGGCTACTT	GGGAGCAGCAAGGGGTCTA
Mus-*Il-6*	TAGTCCTTCCTACCCCAATTTCC	TTGGTCCTTAGCCACTCCTTC
Mus-*Ucp1*	GCTTTGCCTCACTCAGGATTGG	CCAATGAACACTGCCACACCTC
Mus-*Ppara*	ACCACTACGGAGTTCACGCATG	GAATCTTGCAGCTCCGATCACAC
Mus-*Pgc1a*	GAATCAAGCCACTACAGACACCG	CATCCCTCTTGAGCCTTTCGTG
Mus-*Prdm16*	ATCCACAGCACGGTGAAGCCAT	ACATCTGCCCACAGTCCTTGCA
Mus-*Pckl*	GTCTGGCTAAGGAGGAAGGG	CAATGTCATCGCCCACACAT
Mus-*G6p*	GGAGTCTTGTCAGGCATTGCTG	AAGTCCACAGGAGGTCCACCC
Mus-*Fbpl*	TGGTTCCGATGGACACAAGG	CCAATGTGACTGGGGATCAAG
Mus-*Pcx*	TTCTGGGGCCAATGACCTC	TTATACTCCAGACGCCGGAC
Mus-*Gck*	TTACACTGGCCTCCTGATGG	TTTGCAACACTCAGCCAGAC
Mus-*β-actin*	CATTGCTGACAGGATGCAGAAGG	TGCTGGAAGGTGGACAGTGAGG

IL-6, interleukin-6; KLF7, Krüppel-like factor 7; UCP1, uncoupling protein 1.

### Chromatin immunoprecipitation

Chromatin immunoprecipitation (ChIP) for the transcription factor CREB and the *KLF7* promoter was performed using HEK-293T cells according to the SimpleChIP Enzymatic Chromatin IP Kit (CST, 9002). Cross-linking with formaldehyde was performed using 1 × 10^7^ cells, and the chromatin was then fragmented into lengths of 200–1,000 bp. Fragmented chromatin was incubated with an anti-CREB antibody or IgG for immunoprecipitation, and quantitative real-time polymerase chain reaction was conducted with specific primers to assess the relative enrichment of target genes. The sequences of primers used are listed in Table 2.

**Table 2:** Primer for ChIP assay

Primer name	Forward	Reverse
Primer 1	AGGACAAAACAAAAGAGGATTGCTT	AAATTCCACCCAGCTCTCGT
Primer 2	TCAAAGCACTCAAGTAAGCATCC	CCACAAGTGCTCAATGATGAGT
Primer 3	ACTGACACTTTAGGTGCTGAC	AATGCTCTGTCCAACCTTAGAATG
Primer 4	TGTTCTTCATAGAGGATACTGACAC	TGCTCTGTCCAACCTTAGAATG

ChIP, chromatin immunoprecipitation.

### Dual-luciferase reporter assay

The CREB plasmid was transfected with the pGL-3 basic-*KLF7* full-length and truncated forms of the promoter into HEK-293T cells for 48 h. A dual-luciferase reporter assay was performed using the Dual-Luciferase Reporter Assay System (Promega, E1910) according to the manufacturer’s instructions. Briefly, the Renilla luciferase intensity was measured following cell lysis, and the relative response ratio was calculated relative to the control reporter.

### Glucose metabolic tolerance tests

For the glucose tolerance test (GTT), mice were fasted for 16 h but had free access to water. Prior to the assay, the mice were carefully weighed, and initial blood glucose values were obtained. The glucose dose was calculated on the basis of the body weight of the mice (2 g/kg) and then administered through intraperitoneal injection. Blood glucose levels were measured at 30, 60, 90, and 120 min.

For the insulin tolerance test, mice were fasted for 6 h. Insulin was injected intraperitoneally according to body weight (0.5 U/kg), and blood glucose levels were measured in accordance with the GTT protocol.

The protocol for the pyruvate tolerance test was similar to that of the GTT, except that the mice were injected intraperitoneally with 2 g/kg sodium pyruvate.

### Quantification of IL-6

IL-6 levels in serum and cell culture medium were measured using a mouse IL-6 ELISA kit (Proteintech, KE10007, China) following the manufacturer's instructions. The samples and standards were incubated with a precoated enzyme-labeled plate for 2 h. Then, IL-6 bound to the plate was allowed to bind to the detected antibody at 37°C for 1 h. Finally, HRP-labeled streptavidin was added to each well, followed by incubation at 37°C for 40 min for color development. The absorbance values of each well at a wavelength of 450 nm were measured using an enzyme marker and calculated in accordance with the provided instructions.

### Oil red O staining

Differentiated brown adipocytes were washed with PBS and fixed with 4% paraformaldehyde (Servicebio, G1101, China) for 8–10 min at room temperature. After the cells were covered with 60% isopropanol for 15–20 s, oil red O working solution (Servicebio, G1101, China) was added to the cells, which were subsequently incubated at room temperature for 30 min. The cells were rapidly differentiated with 60% isopropanol for 3–5 s and then washed three times with pure water. Stained brown adipocytes were observed and photographed under a microscope.

### Statistical analysis

Statistical analyses were performed using SPSS 26.0 and GraphPad Prism 8.0. Data normality was assessed using the Shapiro–Wilk test. For two-group comparisons, Student’s *t*-tests or Mann-Whitney U tests were applied as appropriate. For experiments involving more than two groups or multiple factors, one-way or two-way ANOVA was used to account for multiple comparisons. Data are presented as dot plots with mean ± SD. Statistical significance was defined as ∗ *P* < 0.05, ∗∗*P* < 0.01, and ∗∗∗*P* < 0.001.

## Results

### Acute stress induces KLF7 and IL-6 in BAT by activating ADRB3

**Fig. 1:**
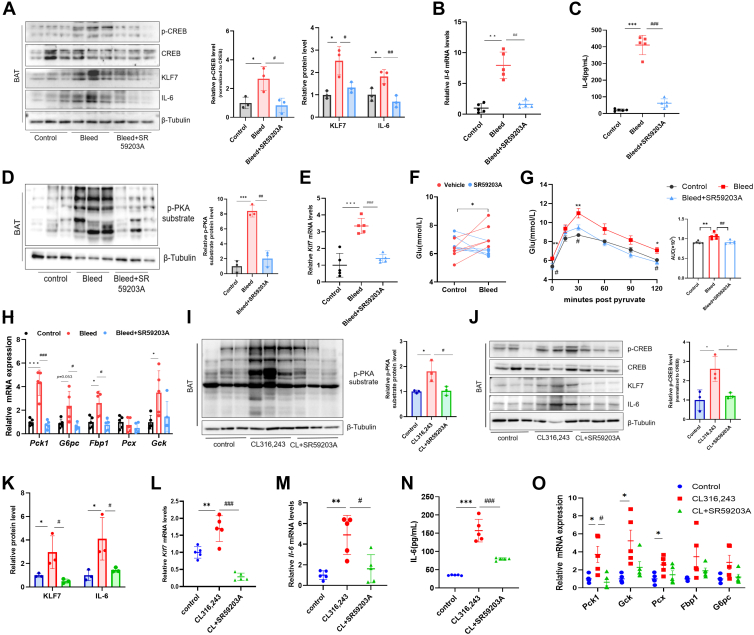
Stress induces KLF7 and IL-6 in BAT by activating ADRB3. A: Immunoblotting for p-CREB, CREB, KLF7, and IL-6 protein levels in the BAT of mice 4 h after retro-orbital bleeding. Quantification of band intensity was performed by densitometric analysis, normalized to β-Tubulin. Band intensities for p-CREB were normalized to total CREB. B: mRNA levels of the *Il-6* gene in the BAT of mice. C: Plasma IL-6 levels in mice after bleeding and injection of the ADRB3 antagonist SR59203A. D: Immunoblotting for p-PKA substrates in the BAT of mice. E: *Klf7* mRNA expression in the BAT of mice after bleeding. F: Blood glucose levels of mice after bleeding. G: PTT was performed in mice 4 h after bleeding. AUC, area under the curve. H: Gluconeogenesis-associated gene expression in the liver of mice after bleeding. I: Protein levels of the p-PKA substrate in mice 2 h post injection of the ADRB3 agonist CL316,243 and the ADRB3 antagonist SR59203A. J: Immunoblotting for p-CREB, CREB, KLF7, and IL-6. K: Quantification of KLF7 and IL-6 protein expression levels by grayscale value analysis. L and M: mRNA levels of *Klf7* and *Il-6* in mice 2 h after administration of CL316,243 and SR59203A. N: Circulating IL-6 levels in mice postinjection. O: mRNA levels of gluconeogenesis-associated genes in the liver. n = 5 per group. Western blot quantification represents densitometric analysis from three independent biological samples. The data are presented as means ± SD. ∗*P* < 0.05, ∗∗*P* < 0.01, and ∗∗∗*P* < 0.001. BAT, brown adipose tissue; IL-6, interleukin-6; KLF7, Krüppel-like factor 7; PKA, protein kinase A; CREB, cAMP response element-binding protein; PTT, pyruvate tolerance test.

Because ADRB3-dependent IL-6 from BAT was identified as the dominant cytokine induced by stress alone (5), we first conducted studies in an acute stress mouse model via retro-orbital bleeding. The levels of IL-6 increased in BAT and serum 4 h after bleeding (Fig. 1A–C). On the basis of our previous findings that KLF7 positively regulates IL-6 in the inflammatory response of white adipocytes (11, 12, 13), we hypothesized that it also plays a role in IL-6 production during stress; therefore, we examined KLF7 induction in BAT after bleeding mice. We found that p-PKA substrate, CREB phosphorylation, and KLF7 were robustly induced in BAT (Fig. 1A, D, E). IL-6 is produced in an ADRB3-dependent manner in BAT (5); therefore, we sought to determine whether ADRB3 is also necessary for KLF7 expression. The ADRB3-specific antagonist SR59203A (5 mg/kg) was applied in conjunction with retro-orbital bleeding. Consistent with the results of previous studies demonstrating that stress induces IL-6 production in an ADRB3-dependent manner (5), SR59203A decreased the stress-induced high levels of IL-6 in BAT and the circulation (Fig. 1A–C), and we also found that pretreatment with SR59203A negated stress-induced p-PKA substrate, CREB phosphorylation and KLF7 expression (Fig. 1A, D, E). Taken together, these findings suggest that, consistent with stress-induced endocrine IL-6, acute stress also increases KLF7 in BAT by activating ADRB3.

Given that stress hyperglycemia is dependent on IL-6, IL-6 originating from brown adipocytes acts on the IL-6 receptor subunit alpha (IL-6 Ra) in the liver to induce hepatic gluconeogenesis (5). In this study, we also assessed glucose metabolism in acutely stressed mice. We found that stress induced an increase in blood glucose (Fig. 1F) but did not affect the GTT or insulin tolerance test results (supplemental Fig. S1A, B); however, pyruvate utilization increased, a finding that is consistent with the results of a previous study (Fig. 1G). We then examined the transcription of gluconeogenesis-related genes, including *Pck1*, *G6pc*, *Fbp1*, *Pcx*, and *Gck* in the liver and kidney, which are pivotal organs for gluconeogenesis, and found that stress upregulated the expression of these genes in the liver (Fig. 1H) but not in the kidney (supplemental Fig. S1C). Furthermore, pretreatment with the ADRB3 antagonist SR59203A prevented stress-induced hyperglycemia elicited by gluconeogenesis in the liver (Fig. 1F–H).

To test the role of ADRB3 in stress-induced KLF7 expression, mice were treated with 1 mg/kg CL316,243 (a specific ADRB3 agonist) for two hours. As anticipated, treatment with CL316,243 activated cAMP/PKA signaling (Fig. 1I, J) and simultaneously increased the transcription and expression of KLF7 and IL-6 in BAT (Fig. 1J–M). CL316,243 increased the levels of circulating IL-6 (Fig. 1N) and transcriptionally induced gluconeogenic genes in the liver (Fig. 1O). Importantly, these effects were reversed by SR59203A (Fig. 1I–O), suggesting that stress induces KLF7 and IL-6 by activating ADRB3.

### ADRB3 activation increases KLF7 and IL-6 expression in brown adipocytes

**Fig. 2:**
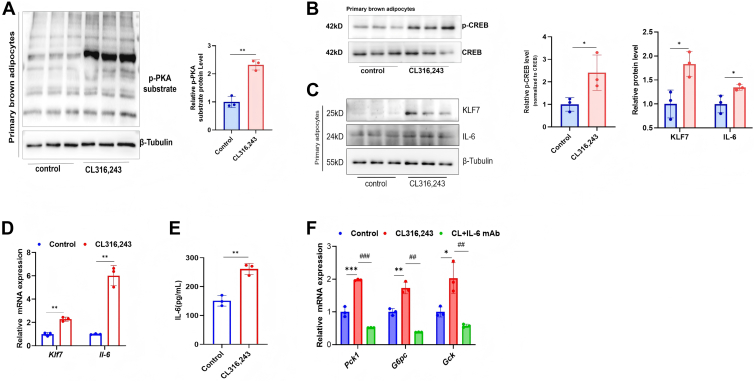
ADRB3 activation increases KLF7 and IL-6 expression in brown adipocytes. A: Phosphorylation of the PKA substrate in primary brown adipocytes treated with 5 μM CL316,243, normalized to β-Tubulin. B: CREB phosphorylation in brown adipocytes, normalized to total CREB. C: Immunoblotting and densitometric quantification of KLF7 and IL-6 protein levels in CL316,243-treated primary brown adipocytes. D: mRNA expression of *Klf7* and *Il-6*. E: IL-6 levels in the medium. F: Gluconeogenesis-associated gene expression in Hepa 1–6 cells cultured with conditioned medium from primary brown adipocytes treated with CL316243. Data are shown as mean ± SD from three independent biological experiments, each performed using independently isolated and differentiated primary brown adipocytes. ∗*P* < 0.05, ∗∗*P* < 0.01, and ∗∗∗*P* < 0.001. IL-6, interleukin-6; KLF7, Krüppel-like factor 7; PKA, protein kinase A; CREB, cAMP response element-binding protein.

To investigate the mechanism by which ADRB3 activation in brown adipocytes induces IL-6 expression, we exposed C3H10T1/2 cells and primary brown adipocytes to CL316,243. After 8 days of differentiation, mature brown adipocytes exhibited significant small lipid droplet accumulation (supplemental Fig. S2A). We examined thermogenesis markers to characterize brown adipocytes and detected increased UCP1 expression and thermogenesis-related gene transcription in differentiated brown adipocytes (supplemental Fig. S2B–E), indicating that brown adipocytes were successfully differentiated. Our data suggest that, consistent with changes in acutely stressed mice, 5 μM CL316,243 treatment for 4 h induced PKA substrate and CREB phosphorylation (Fig. 2A, B and supplemental Fig. S2F, G) and KLF7 and IL-6 expression in brown adipocytes (Fig. 2C, D and supplemental Fig. S2H, I). Concurrently, we found that compared with the saline control, the ADRB3 agonist CL316,243 increased the level of IL-6 in the medium (Fig. 2E and supplemental Fig. S2J). CL316,243-treated brown adipocyte medium was harvested from cell culture plates and cocultured with mouse hepatocytes for 4 h, resulting in the transcription of genes associated with hepatic gluconeogenesis; however, anti-IL-6 neutralizing antibodies ablated this effect (Fig. 2F and supplemental Fig. S2K). Taken together, these data indicate that the ADRB3 agonist CL316,243 activates cAMP/PKA signaling and increases the expression of KLF7 and IL-6 in brown adipocytes. Importantly, brown adipocyte-derived IL-6 contributes to the upregulation of gluconeogenic gene expression in hepatocytes.

### ADRB3-induced IL-6 expression requires KLF7 in adipocytes during stress

**Fig. 3:**
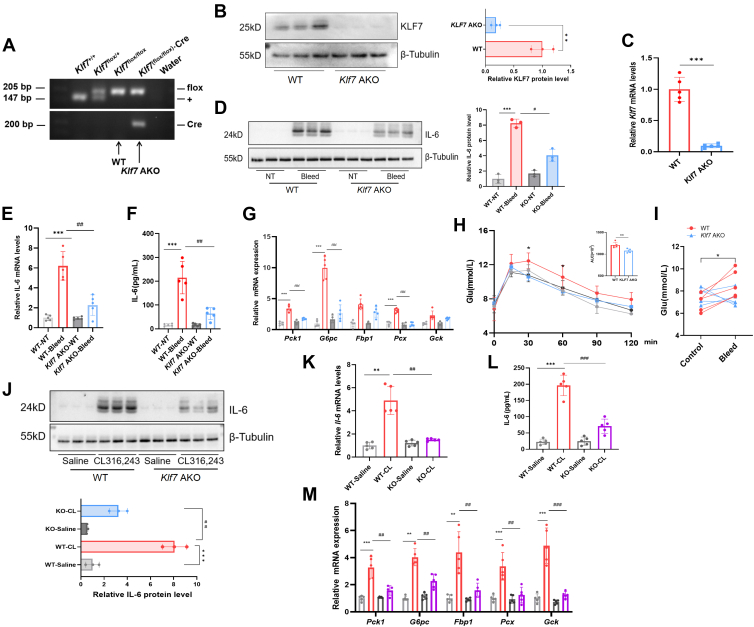
ADRB3-induced IL-6 expression requires *Klf7* in adipocytes during stress. A: Representative genotyping results for WT and *Klf7* AKO mice. B: Representative western blots and densitometric quantification showing KLF7 levels in the BAT of WT and *Klf7* AKO mice. C: *Klf7* mRNA levels in the BAT of WT and *Klf7* AKO mice. D: The levels of IL-6 in *Klf7* AKO or WT mice after bleeding. E: mRNA expression of IL-6 in BAT. F: Circulating IL-6 levels. G: Gluconeogenic gene expression in the livers of WT and *Klf7* AKO mice. H: PTT was performed in mice after bleeding. I: Blood glucose levels in mice after bleeding (n = 5 per group). J: Protein levels of IL-6 in mice 2 h after administration of CL316,243. K: mRNA expression in the BAT of mice after treated with 5 mg/kg CL316,243. L: Circulating IL-6 levels. M: Gluconeogenesis-associated gene expression in the livers of WT and *Klf7* AKO mice. NT, no treated. n = 5 per group. Western blot quantification represents densitometric analysis from three independent biological samples. The data are presented as means ± SD. ∗*P* < 0.05, ∗∗*P* < 0.01, and ∗∗∗*P* < 0.001. BAT, brown adipose tissue; IL-6, interleukin-6; KLF7, Krüppel-like factor 7; *Klf7* AKO, *Klf7*-adipocyte knockout; PTT, pyruvate tolerance test.

To determine whether KLF7 is essential for brown adipocyte-derived IL-6 production during stress, we generated *Klf7*-adipocyte knockout (*Klf7* AKO) mice and stressed them via retro-orbital bleeding (Fig. 3A‒C). We found that *Klf7* deletion decreased IL-6 levels in BAT and the circulation after bleeding (Fig. 3D–H), whereas there were no differences in p-PKA substrates or p-CREB between *Klf7* AKO and WT littermates (supplemental Fig. S3A, B). In addition, *Klf7* AKO mice presented lower transcription of *Pck1*, *G6pc*, *Pcx*, and other gluconeogenesis-associated genes in the liver (Fig. 3I). Pyruvate tolerance tests were performed using *Klf7* AKO and WT mice after bleeding, and our data suggest that the capacity for pyruvate utilization was diminished in stressed *Klf7* AKO mice (Fig. 3J), resulting in lower blood glucose levels after 4 h of acute stress (Fig. 3K). Thus, these data indicate that stress-induced IL-6 expression in adipocytes requires *Klf7*.

Consistent with the observations in mice stressed via retro-orbital bleeding, we did not detect any differences in PKA substrate and CREB phosphorylation after the administration of 5 mg/kg CL316,243 for 2 h (supplemental Fig. S3C, D); however, we found that a lack of *Klf7* greatly reduced the expression of CL316,243-induced IL-6 in BAT (Fig. 3L, M), as well as circulating IL-6 levels (Fig. 3N). In addition, the transcription of genes related to hepatic gluconeogenesis was also inhibited (Fig. 3O). Collectively, these data indicate that the activation of ADRB3-induced IL-6 production requires *Klf7* in adipocytes during acute stress.

### Stress-induced upregulation of KLF7 and IL-6 in BAT is mediated by PKA activation

**Fig. 4:**
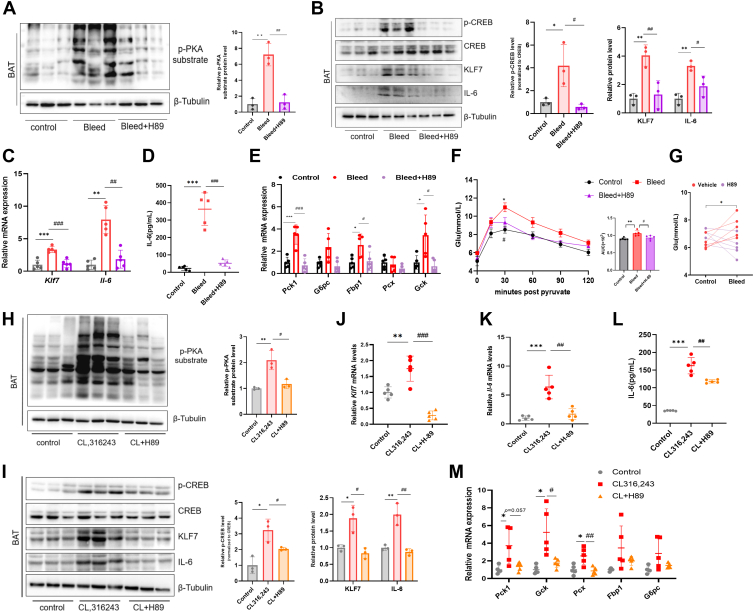
Stress-induced upregulation of KLF7 and IL-6 in BAT is mediated by PKA activation. A: Representative western blots and densitometric quantification of PKA substrate phosphorylation after bleeding and administration of the PKA inhibitor H89. B: p-CREB, CREB, KLF7, and IL-6 protein levels in BAT. C: *Klf7* and *Il-6* mRNA levels in the BAT of mice 4 h after retro-orbital bleeding and the injection of the PKA inhibitor H89. D: Plasma IL-6 levels. E: Gluconeogenesis-associated gene expression in the liver of mice after bleeding. F: PTT was performed in mice 4 h after bleeding. G: Blood glucose levels in mice after bleeding. n = 5 per group. H and I: PKA substrate and CREB phosphorylation in the BAT of mice after the injection of CL316,243 and H89. J and K: mRNA and protein levels of *Klf7* and *Il-6*. L: Plasma levels of IL-6. M: mRNA levels of gluconeogenic genes in the liver. n = 5 per group. Western blot quantification represents densitometric analysis from three independent biological samples. The data are presented as means ± SD. ∗*P* < 0.05, ∗∗*P* < 0.01, and ∗∗∗*P* < 0.001. BAT, brown adipose tissue; IL-6, interleukin-6; KLF7, Krüppel-like factor 7; PKA, protein kinase A; CREB, cAMP response element-binding protein; PTT, pyruvate tolerance test.

To examine whether cAMP/PKA signaling mediates the stress-induced upregulation of KLF7 and IL-6 expression in BAT, we pretreated mice with the PKA inhibitor H89 (5 mg/kg) before bleeding. Compared with vehicle treatment, H89 administration effectively blocked PKA activity in the BAT of mice after bleeding (Fig. 4A). We found that H89 inhibited the phosphorylation of the transcription factor CREB, decreased stress-induced KLF7 and IL-6 expression in BAT (Fig. 4B, C), and decreased circulating IL-6 levels (Fig. 4D), suggesting that the stress-induced expression of KLF7 and IL-6 in BAT is dependent on PKA activation. We subsequently investigated the effect of the PKA inhibitor H89 on IL-6-induced hyperglycemia during stress, and the results revealed that H89 blunted the effects of stress-induced liver gluconeogenesis (Fig. 4E) and pyruvate utilization (Fig. 4F), leading to a decrease in blood glucose levels (Fig. 4G). Taken together, these results indicate that blocking PKA suppresses the stress-induced expression of KLF7 and IL-6 in BAT and inhibits hepatic gluconeogenic gene expression.

We next examined whether PKA is a prerequisite mediator of ADRB3-induced KLF7 and IL-6 expression. Similar to observations in stressed mice, pretreatment with 5 mg/kg H89 (a PKA blocker) adequately reduced CL316,243-induced PKA activity in BAT (Fig. 4H). Moreover, compared with those in Cl316243-treated mice, H89-treated mice exhibited lower levels of the PKA downstream signaling factor p-CREB (Fig. 4I), reduced KLF7 expression in BAT (Fig. 4I, J), and decreased IL-6 levels both in BAT and in circulation (Fig. 4I–L). Furthermore, H89 reversed the CL316,243-induced transcription of *Pck1* and other gluconeogenesis-related genes in the liver (Fig. 4M), demonstrating that PKA plays a crucial role in the upregulation of KLF7 and IL-6 expression induced by ADRB3 activation and gluconeogenic gene expression in the liver.

### PKA mediates the upregulation of KLF7 and IL-6 expression induced by ADRB3 activation in brown adipocytes

**Fig. 5:**
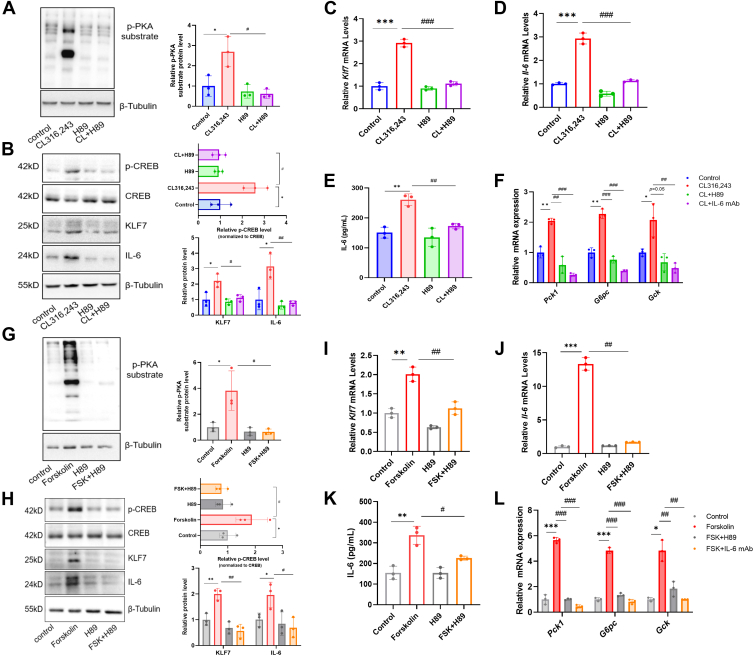
PKA mediates the upregulation of KLF7 and IL-6 expression induced by ADRB3 activation and cAMP elevation in brown adipocytes. A: p-PKA substrate levels in primary brown adipocytes treated with CL316,243 and H89, normalized to β-tubulin. B: p-CREB, CREB, KLF7, and IL-6 protein levels in H89-pretreated primary brown adipocytes, normalized to β-tubulin. Band intensities for p-CREB were normalized to total CREB. C and D: *Klf7* and *Il-6* mRNA expression. E: IL-6 levels in the medium of primary brown adipocytes treated with the indicated compounds (5 μM CL316243 or 10 μM H89). F: mRNA levels of gluconeogenesis-associated genes in Hepa 1–6 cells cultured with conditioned medium from primary brown adipocytes treated with the indicated compounds (5 μM CL316243, 10 μM H89, or 2 μg/ml anti-IL-6 mAb). G: p-PKA substrate levels in primary brown adipocytes treated with 10 μM forskolin and 10 μM H89. H: p-CREB, CREB, KLF7, and IL-6 protein expression in primary brown adipocytes. I: mRNA expression of *Klf7* in forskolin- and H89-treated primary brown adipocytes. J: *Il-6* mRNA levels in primary brown adipocytes. K: IL-6 levels in the medium of brown adipocytes. L: Gluconeogenesis-associated gene expression levels in Hepa 1–6 cells cultured with conditioned medium from primary brown adipocytes treated with the indicated compounds (10 μM forskolin, 10 μM H89, or 2 μg/ml anti-IL-6 mAb). Data are shown as mean ± SD from three independent biological experiments, each performed using independently isolated and differentiated primary brown adipocytes. ∗*P* < 0.05, ∗∗*P* < 0.01, and ∗∗∗*P* < 0.001. IL-6, interleukin-6; KLF7, Krüppel-like factor 7; PKA, protein kinase A; CREB, cAMP response element-binding protein.

Stress-induced IL-6 originates from brown adipocytes, as identified in UCP1 promoter-driven IL-6 knockout mice (5); thus, we investigated the regulatory relationship between ADRB3 and PKA in brown adipocytes in vitro. We found that treatment with the ADRB3 agonist CL316,243 induced PKA substrate phosphorylation in primary brown adipocytes and C3H10T1/2 cells, whereas pretreatment with the PKA inhibitor H89 (5 μM) prevented this effect (Fig. 5A and supplemental Fig. S4A). We also observed that H89 reduced the phosphorylation of CREB (Fig. 5B and supplemental Fig. S4B) and inhibited the CL316,243-induced transcription and expression of KLF7 and IL-6 in brown adipocytes (Fig. 5B–D and supplemental Fig. S4B–D). Moreover, pretreatment with H89 also suppressed the release of IL-6 induced by ADRB3 activation in primary brown adipocytes and C3H10T1/2 cells (Fig. 5E and supplemental Fig. S4E). After 4 h of coculture with medium from brown adipocytes treated with CL316,243, the transcription of the gluconeogenic genes *Pck1*, *G6pc*, and *Gck* increased in Hepa 1–6 mouse hepatocytes (Fig. 5F and supplemental Fig. S4F); however, the culture medium from brown adipocytes treated with CL316,243 and H89 reduced their transcription, approaching the effect of an anti-IL-6 neutralizing antibody (Fig. 5F and supplemental Fig. S4F), demonstrating that the activation of ADRB3 in brown adipocytes results in the upregulation of KLF7 and IL-6 expression through PKA signaling.

### cAMP enhances the expression of KLF7 and IL-6 via PKA in brown adipocytes

The activation of PKA is contingent upon the concentration of cAMP, a second messenger elicited by adenylate cyclase; therefore, we investigated the pivotal role of cAMP in ADRB3-induced IL-6 expression. Forskolin, a compound that increases the concentration of cAMP, was introduced into the medium of brown adipocytes. Our findings indicated that, compared with vehicle, forskolin effectively stimulated PKA and led to an increase in CREB phosphorylation (Fig. 5G, H and supplemental Fig. S4G, H). The transcription and expression of KLF7 and IL-6 were also found to be upregulated by forskolin (Fig. 5H–J and supplemental Fig. S4H–J), and IL-6 accumulated in the medium (Fig. 5K and supplemental Fig. S4K), suggesting that cAMP is able to activate PKA and increase KLF7 and IL-6 levels in brown adipocytes. In addition, the medium of brown adipocytes treated with forskolin increased the transcription of the gluconeogenic genes *Pck1*, *G6pc*, and *Gck* in hepatocytes and an anti-IL-6 neutralizing antibody blocked this effect (Fig. 5L and supplemental Fig. S4L). To investigate the contribution of PKA to cAMP-induced IL-6 expression, we pretreated brown adipocytes with the PKA inhibitor H89 before the administration of forskolin, and our data revealed that the effects elicited by forskolin were effectively nullified by H89 (Fig. 5 and supplemental Fig. S4). In summary, our findings demonstrate that cAMP increases the phosphorylation of CREB and promotes the expression of KLF7 and IL-6 by activating PKA in brown adipocytes.

### CREB mediates ADRB3-induced KLF7 and IL-6 expression in brown adipocytes and transcriptionally activates KLF7

**Fig. 6:**
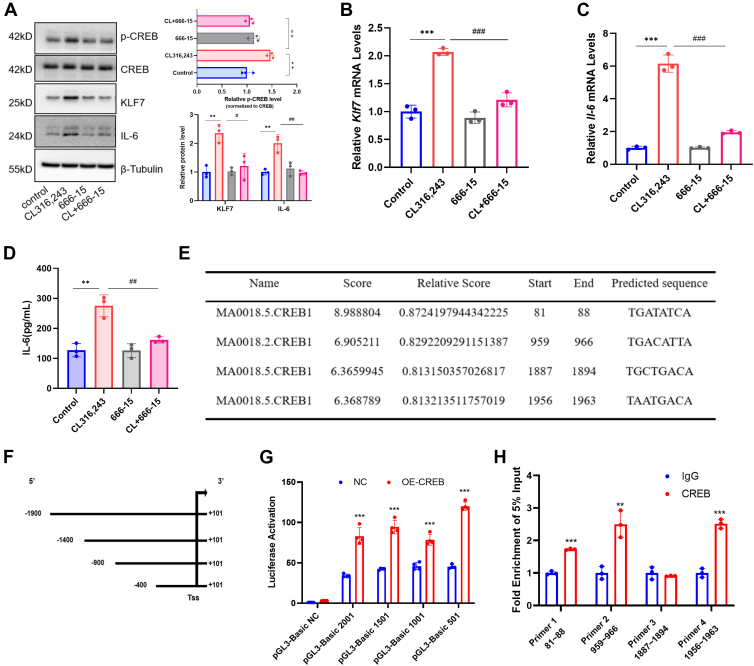
CREB mediates ADRB3-induced KLF7 and IL-6 expression in brown adipocytes and transcriptionally activates KLF7. A: Immunoblotting for p-CREB, CREB, KLF7, and IL-6 in 666-15-pretreated primary brown adipocytes, normalized to β-tubulin. Band intensities for p-CREB were normalized to total CREB. B: *Klf7* mRNA levels. C: *Il-6* mRNA levels. D: IL-6 levels in the medium of primary brown adipocytes treated with the indicated compounds (5 μM CL316,243 and 10 μM 666-15). E: Binding site of CREB to KLF7 as predicted by the JASPAR database. F: Schematic of *KLF7* fluorescent plasmids. G: *KLF7* fluorescence activity values in CREB-overexpressing HEK-293T cells. H: ChIP assay performed in CREB-overexpressing HEK-293T cells. Data are shown as mean ± SD from three independent biological experiments performed using independently prepared primary brown adipocytes or HEK-293T cells. ∗*P* < 0.05, ∗∗*P* < 0.01, and ∗∗∗*P* < 0.001. IL-6, interleukin-6; KLF7, Krüppel-like factor 7; CREB, cAMP response element-binding protein; ChIP, chromatin immunoprecipitation.

To further validate the functional role of CREB in the ADRB3-KLF7-IL-6 signaling axis, we employed the CREB inhibitor 666-15 to interrogate whether CREB activity is necessary for ADRB3-induced IL-6 production in brown adipocytes. Primary brown adipocytes were pretreated with 666-15 for 1 h, followed by stimulation with the ADRB3 agonist CL316,243 for 4 h. We observed that 666-15 suppressed CL316,243-induced CREB phosphorylation (Fig. 6A). Consequently, the upregulation of both KLF7 and IL-6 at the mRNA and protein levels by CL316,243 was markedly attenuated in the presence of 666-15 (Fig. 6A–C). Consistent with these findings, the secretion of IL-6 into the culture medium was also significantly reduced upon CREB inhibition (Fig. 6D). These data collectively demonstrate that CREB activation is indispensable for ADRB3-triggered KLF7 expression and subsequent IL-6 production in brown adipocytes.

Given that CREB is a pivotal transcription factor with the capacity to regulate an extensive array of target genes, we hypothesized that CREB is involved in the transcription of KLF7. To test this hypothesis, we queried the promoter sequence of *KLF7* via the NCBI database and then performed predictive analysis with the transcription factor CREB in the JASPAR database. After applying an 80% score threshold for screening and considering overlapping binding sites, we identified a total of four binding sites located within the *KLF7* promoter region that interact with CREB (Fig. 6E). To examine the targeted regulatory relationship between CREB and *KLF7*, we generated three truncated forms (−400, −900, and −1,400 relative to the transcription start site) of the *KLF7* promoter and inserted them into luciferase plasmids for dual-luciferase reporter gene assays (Fig. 6F). Following cotransfection of the CREB plasmid into HEK-293T cells for 48 h, we observed that overexpression of CREB increased the luciferase activity of the full-length *KLF7* promoter, as well as the activity of the three truncated promoters (Fig. 6G). Four specific primers targeting *KLF7* were designed on the basis of the predicted binding sites, and ChIP assays were performed using HEK-293T cells transfected with the CREB plasmid, revealing that CREB binds to the −88∼-81, −966∼-959, and −1963∼-1956 sites within the promoter region of *KLF7* (Fig. 6H). Thus, our data confirmed that the transcription factor CREB binds to the promoter of KLF7 and positively regulates its transcription.

## Discussion

Numerous studies have confirmed that acute stress induces the release of IL-6 in human, rat, and mouse models (19, 20, 21); however, the origin of IL-6 and its correlation with organismal metabolism during acute stress remain unclear. A recent investigation by Qing *et al.* revealed that BAT releases most of endocrine IL-6 in an ADRB3-dependent manner during stress and that IL-6 enhances liver gluconeogenesis by acting on the IL-6 receptor in the liver (5). Nevertheless, the molecular mechanism underlying the release of IL-6 following ADRB3 activation in brown adipocytes during stress has not been well studied. In this study, our data define KLF7 as a necessary mediator and suggest it may be a sufficient mediator of ADRB3-induced IL-6 production from brown adipocytes during stress; in addition, we determined that ADRB3 positively regulates KLF7 transcription via cAMP/PKA/CREB signaling.

Although BAT is canonically regarded as a thermogenic organ, accumulating evidence indicates that its endocrine outputs can be dissociated from canonical thermogenesis. Acute psychological stress activates adrenergic signaling in BAT and drives robust endocrine IL-6 release via ADRB3, yet this IL-6 response occurs without induction of the classical thermogenic transcriptional program, is independent of UCP1, and is not altered by ambient temperature, indicating that stress-evoked IL-6 is not simply a byproduct of increased heat production (5). Moreover, BAT can rapidly increase blood flow in ways that are separable from uncoupled mitochondrial respiration, providing an efficient route for hormone release that does not require activation of the full thermogenic machinery. Thus, in the context of acute stress, adipocytes, particularly those in BAT, can function as stress-responsive endocrine cells, and ADRB3-driven IL-6 secretion from these cells serves a metabolic signaling role distinct from the thermogenic program involved in defending body temperature. This functional separation has important implications for interpreting BAT biology in stress versus cold exposure and for considering therapeutic strategies that target adrenergic or IL-6 signaling.

The lipotoxicity caused by increased lipolysis in obesity is known to increase the release of adipocyte-derived IL-6 and other cytokines. In this setting, the exacerbated inflammatory response promptly catalyzes the progression of metabolic diseases associated with obesity, including insulin resistance and type 2 diabetes (22, 23, 24). We have conducted a series of studies to investigate the potential mechanisms of obesity-induced inflammation. Zhang *et al.* demonstrated that palmitic acid induces IL-6 expression by activating TLR4/KLF7/NF-κB signaling in white adipocytes and that KLF7 plays a positive regulatory role in *IL-6* transcription (12). Qiu *et al.* reported that the elevated serum levels of palmitic acid in obesity enhance the phosphorylation of the NF-κB subunit P65 by binding to the fatty acid-specific receptor GPR40/120, which enhances the transcription of *KLF7*, resulting in the increased expression of IL-6 in white adipocytes (25). Yang *et al.* revealed that the transcription factor KLF7 enhances the transcription of *Pkcζ* by binding to its promoter region, subsequently leading to the increased transcription of *P65*, which subsequently activates *IL-6* expression (11). These studies indicate that the positive regulatory loop between P65 and KLF7 plays a crucial role in the inflammatory response of adipocytes, especially the expression of IL-6, which suggests that this loop may initiate brown adipocyte-derived IL-6 production during stress. However, Tchivileva *et al*. revealed that ADRB3-induced IL-6 production is independent of NF-κB signal transduction (26). In this study, in brown adipocytes treated with forskolin and H89, we found that ADRB3 induced KLF7 expression via the cAMP/PKA/CREB pathway and that *Klf7* deletion in adipocytes reversed this effect, suggesting that *Klf7* mediates ADRB3-dependent IL-6 production in adipocytes. In addition, as mentioned above, the activation of TLRs and GPRs induces the expression of KLF7; therefore, whether these pathways mediate stress-induced IL-6 production needs to be further studied.

ADRB3 activation leads to an increase in cAMP levels, which is the result of coupling to Gas. Elevated cAMP levels can directly activate PKA or engage in the cAMP-EPAC pathway to increase downstream cascades. In cold exposure or ADRB3 agonist administration, ADRB3 activation increases lipolysis through the cAMP-PKA pathway, resulting in free fatty acid release, which stimulates the expression of UCP1 in the mitochondrial inner membrane, thereby activating the thermogenic programs of BAT to promote energy expenditure (27, 28). In this study, we observed a significant increase in the levels of PKA substrate and CREB phosphorylation, as well as KLF7 and IL-6 expression, in CL316,243- or forskolin-treated brown adipocytes; importantly, the impact of these effects was counteracted by the administration of the PKA inhibitor H89. We also identified multiple binding sites between the transcription factor CREB and the promoter region of *KLF7*, suggesting that the cAMP-PKA-CREB pathway contributes to the ADRB3-induced activation of KLF7 and IL-6 expression during stress. In addition, ADRB3 affects various processes through the cAMP-EPAC pathway, such as tumor nerve innervation and detrusor and bladder smooth muscle contraction (29, 30, 31). However, whether cAMP/EPAC signaling downstream of ADRB3 is involved in ADRB3-induced KLF7 and IL-6 expression is worthy of further investigation.

CREB senses and responds to extracellular fluctuations in nutrient concentrations, hormone levels and energy balance and functions as a transcription factor or cofactor with a key role in regulating whole-body homeostasis (32, 33). In mammals, thousands of gene promoter regions contain one or more CRE sites. However, the role of CREB in regulating gene expression across various cell types via specific or divergent intracellular signals remains largely unknown. In this study, we demonstrated that CREB positively regulates the transcription of *KLF7* by binding to its promoter, which may contributes to stress-induced IL-6 production. Notably, Saifudeen *et al.* reported that the interaction between KLF4 and CREB affects the expression of the bradykinin B2 receptor during nephron differentiation (34), and Kingsbury *et al.* reported that KLF7 functionally interacts with CREB to promote TRKB P2 transcription in neuronal cells (35), suggesting that there may be functional interactions between CREB and the KLF family. Hence, the functional interplay between CREB and KLF7, and its broader implications for energy metabolism, merit further exploration. Furthermore, Tchivileva *et al.* reported that CREB forms a heterodimer with its family member ATF2, which can activate IL-6 transcription in ADRB3 agonist-treated white adipocytes (26); however, whether this mechanism operates in brown adipocytes under stress conditions needs to be further elucidated.

Gluconeogenesis is typically stimulated by insulin-like growth factor and glucagon in fasting, ketogenic diets or other low-energy states through the activation of key transcription factors such as CREB and FoxOs, which facilitate the expression of gluconeogenesis-related enzymes, including *Pck* and *G6p*, as well as the nuclear receptor coactivator PGC-1α to increase glucose production (36, 37, 38). Unlike the process of glucose synthesis that occurs during starvation, IL-6 is an essential signal indicative of stress-induced liver gluconeogenesis even in net energy-positive states (5). Early investigations demonstrated that primary rat hepatocytes incubated with IL-6 exhibited increased gluconeogenesis (39); the administration of IL-6 also resulted in elevated hepatic glucose production, whereas neutralizing IL-6 reduced glucogenesis in the livers of rats fed a high-fat diet (40). In recent years, Qing *et al.* revealed a similar phenomenon in which stress-induced IL-6 binding to hepatocyte IL-6R stimulates liver gluconeogenesis, which fuels “fight or flight” responses (5). Consistent with these studies, here, we found that IL-6 derived from ADRB3 agonist-treated adipocytes enhanced the transcription of gluconeogenesis-related genes in mouse hepatocytes and that a neutralizing antibody against IL-6 abolished this effect, supporting a role for adipocyte-derived IL-6 in regulating hepatic gluconeogenic gene expression. IL-6 elicits classical or nonclassical signal transduction by binding to either membrane-anchored or soluble IL-6R, which in turn forms complexes with gp130, ultimately resulting in the activation of JAK/STAT and SHP2/Gab/MAPK signaling for either or both (41, 42). However, the mechanism by which stress-induced IL-6 promotes hepatocyte gluconeogenesis is still not entirely clear. Notably, IL-6 membrane-anchored receptors are expressed exclusively in hepatocytes and certain leukocyte subpopulations (43). Studies have also demonstrated that tumor-induced IL-6 promotes gluconeogenic gene expression through liver JAK-STAT signaling in mice and *Drosophila*. Furthermore, the inhibition of glucagon-like hormone signaling has no significant effect on gluconeogenesis, suggesting that the role of IL-6 in liver gluconeogenesis is independent of glucagon (44). Skeletal muscle-derived IL-6 activates STAT3 in the liver during exercise, consequently increasing the transcriptional activity of gluconeogenic genes such as *Pck*. Conversely, IL-6-induced *Pck* expression was significantly inhibited in *STAT3*-knockout mice (45, 46). These studies collectively suggest that IL-6 binds to IL-6R in hepatocytes to activate classical intracellular signal transduction, indicating a potential role for the JAK/STAT3 pathway in stress-induced hepatic gluconeogenesis.

In conclusion, on the basis of previous studies, we explored the specific molecular mechanism of ADRB3-induced IL-6 production in adipocytes during stress, that is, cAMP-PKA-CREB-KLF7 signaling, which increases IL-6 expression and promotes the expression of hepatic gluconeogenic genes. These findings provide a theoretical basis for fully elucidating the specific molecular mechanisms of IL-6 production in adipocytes during stress and further our understanding of the role and function of adipose tissue.

## Ethics Approval and Consent to Participate

All experimental animals in this study were approved by the First Affiliated Hospital of Shihezi University and subjected to regulatory review (No. A2019-086-01).

## Data Availability

The data that support the findings of this study are available in the Materials and Methods, Results, and/or Supplemental Material of this article.

## Supplemental data

This article contains supplemental data.

## Conflict of interest

The author declares that they have no conflicts of interest with the contents of this article.
