# Supplementary figures and images for: KLF7 induced ADRB3-dependent IL-6 production in brown adipocytes during stress
Source: J Lipid Res. 2026 Jan 19;67(2):100981. doi: 10.1016/j.jlr.2026.100981 (PMC12925546; doi:10.1016/j.jlr.2026.100981)

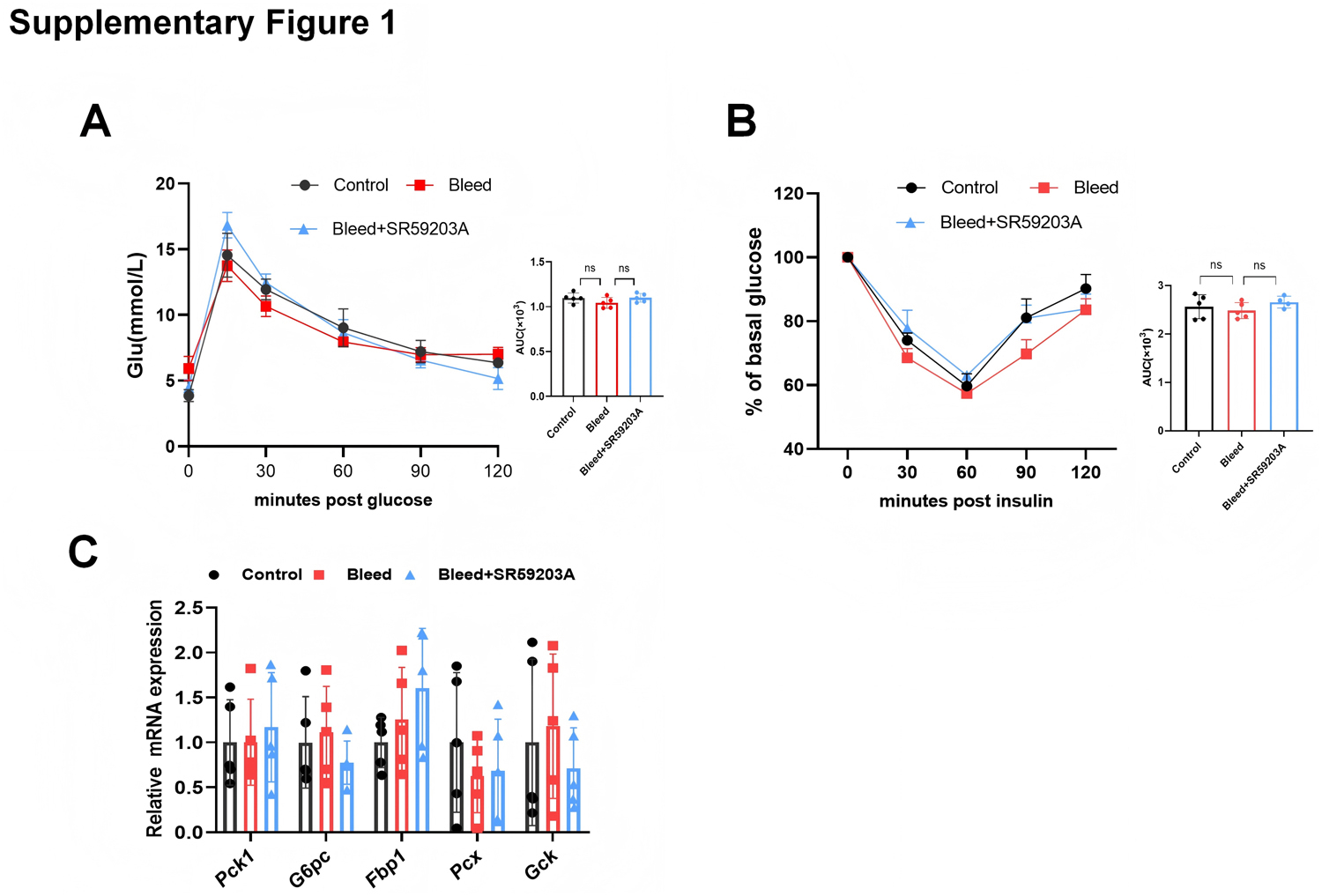

Supplement: Supplementary Figure 1 [file figs1.jpg]

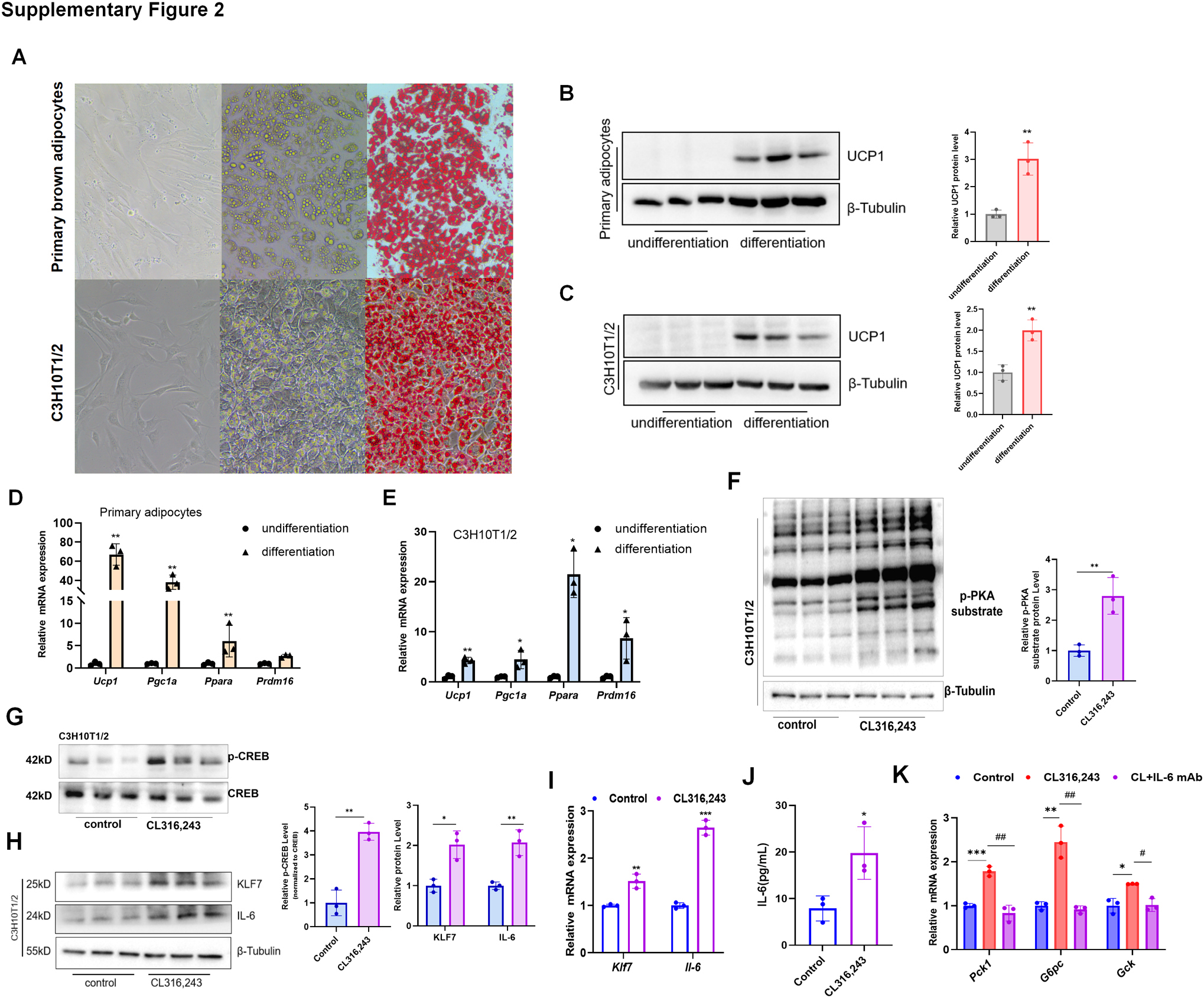

Supplement: Supplementary Figure 2 [file figs2.jpg]

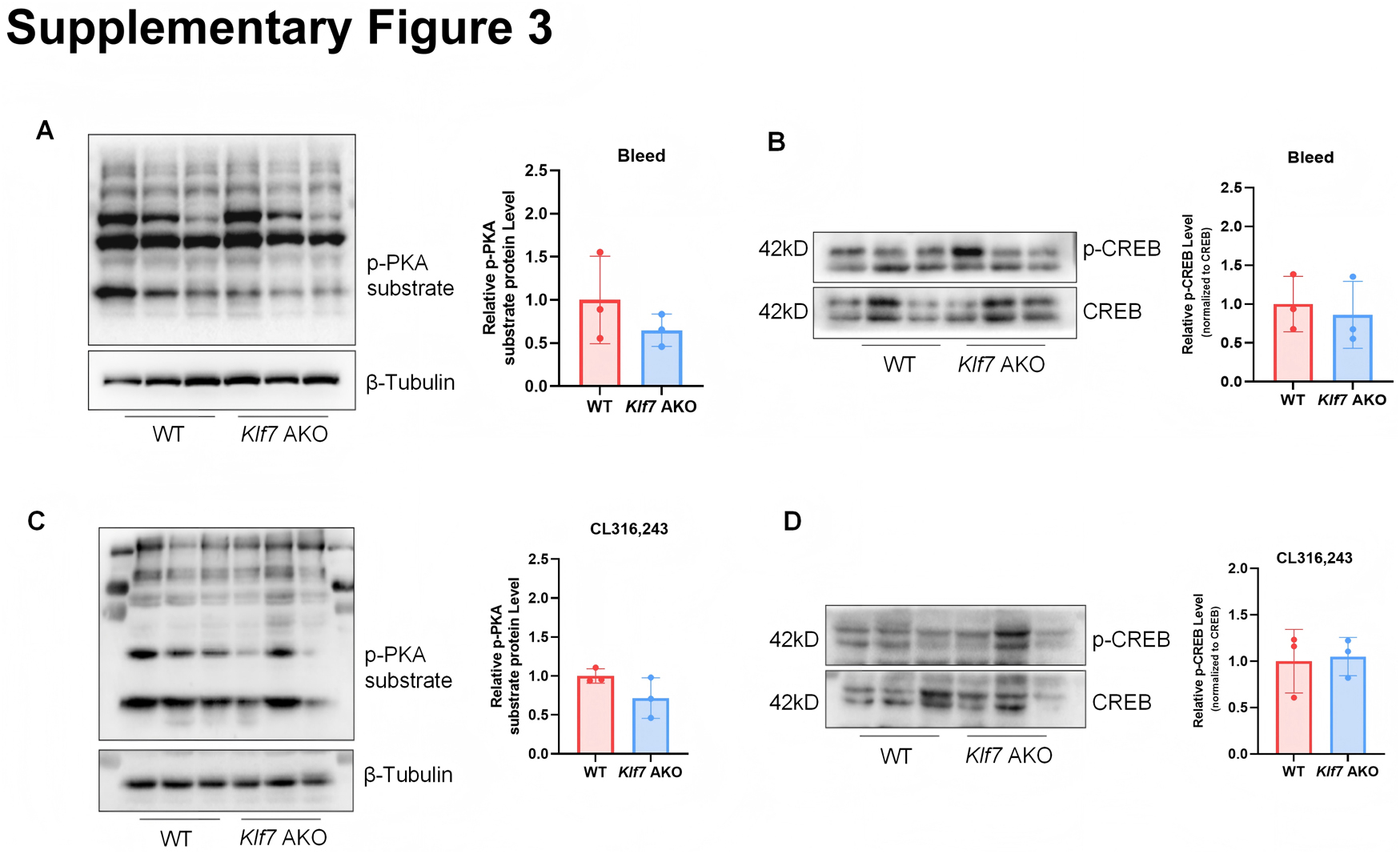

Supplement: Supplementary Figure 3 [file figs3.jpg]

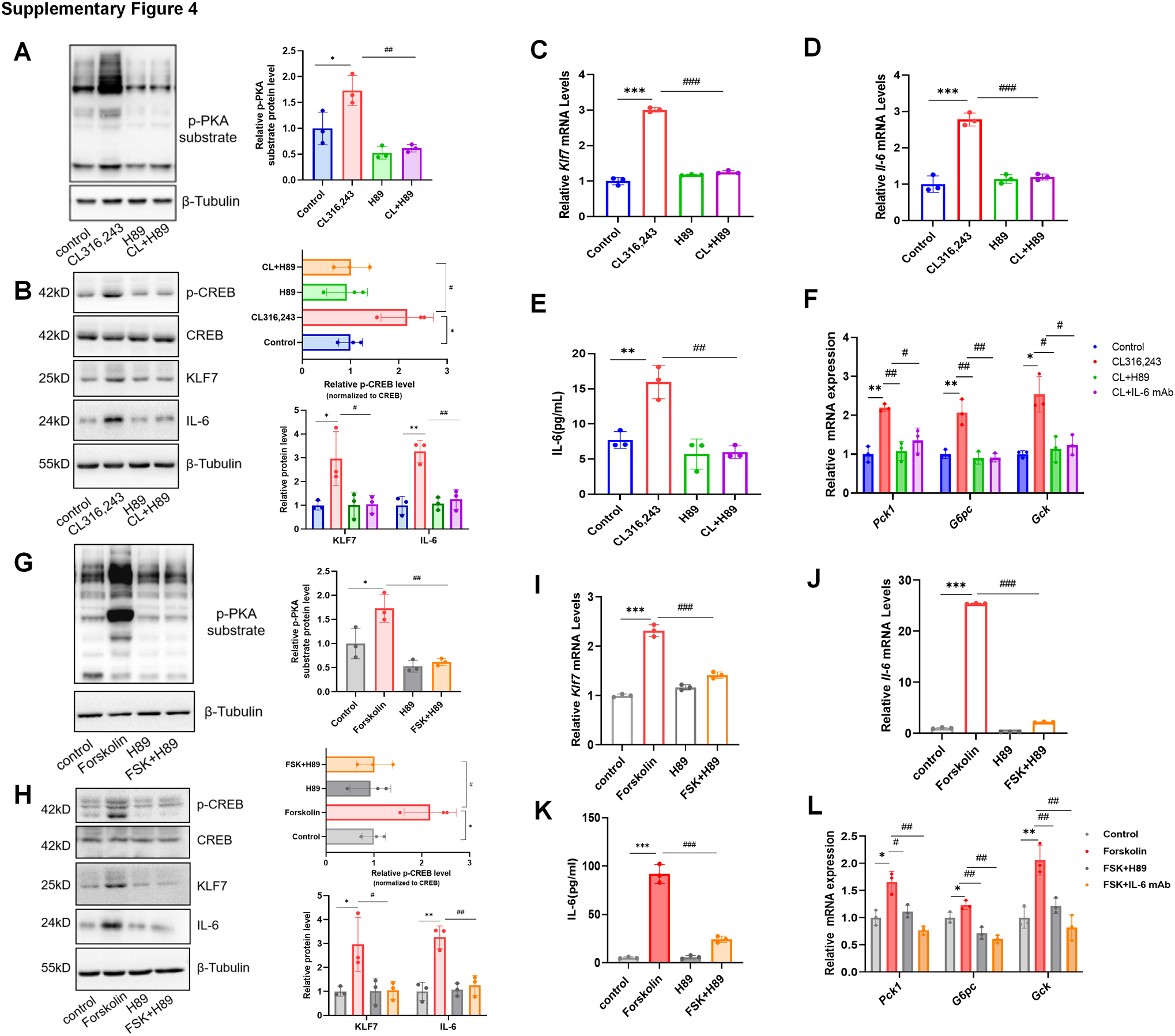

Supplement: Supplementary Figure 4 [file figs4.jpg]
